# WSVAS: A YOLOv4 -based phenotyping platform for automatically detecting the salt tolerance of wheat based on seed germination vigour

**DOI:** 10.3389/fpls.2022.1074360

**Published:** 2022-12-20

**Authors:** Xiuqing Fu, Bing Han, Shouyang Liu, Jiayi Zhou, Hongwen Zhang, Hongbiao Wang, Hui Zhang, Zhiqian Ouyang

**Affiliations:** ^1^ College of Engineering, Nanjing Agricultural University, Nanjing, China; ^2^ Key laboratory of Intelligence Agricultural Equipment of Jiangsu Province, Education Department of Jiangsu Province and is managed by the College of Engineering of Nanjing Agricultural University, Nanjing, China; ^3^ Academy For Advanced Interdisciplinary Studies, Nanjing Agricultural University, Nanjing, China; ^4^ School of Mechanical and Electrical Engineering, Shihezi University, Shihezi, China; ^5^ College of Mechanical and Electrical Engineering, Tarim University, Alar, China

**Keywords:** wheat seeds, salt tolerance, phenotypic platform, convolutional neural network, germination percentage

## Abstract

Salt stress is one of the major environmental stress factors that affect and limit wheat production worldwide. Therefore, properly evaluating wheat genotypes during the germination stage could be one of the effective ways to improve yield. Currently, phenotypic identification platforms are widely used in the seed breeding process, which can improve the speed of detection compared with traditional methods. We developed the Wheat Seed Vigour Assessment System (WSVAS), which enables rapid and accurate detection of wheat seed germination using the lightweight convolutional neural network YOLOv4. The WSVAS system can automatically acquire, process and analyse image data of wheat varieties to evaluate the response of wheat seeds to salt stress under controlled environments. The WSVAS image acquisition system was set up to continuously acquire images of seeds of four wheat varieties under three types of salt stress. In this paper, we verified the accuracy of WSVAS by comparing manual scoring. The cumulative germination curves of wheat seeds of four genotypes under three salt stresses were also investigated. In this study, we compared three models, VGG16 + Faster R-CNN, ResNet50 + Faster R-CNN and YOLOv4. We found that YOLOv4 was the best model for wheat seed germination target detection, and the results showed that the model achieved an average detection accuracy (mAP) of 97.59%, a recall rate (Recall) of 97.35% and the detection speed was up to 6.82 FPS. This proved that the model could effectively detect the number of germinating seeds in wheat. In addition, the germination rate and germination index of the two indicators were highly correlated with germination vigour, indicating significant differences in salt tolerance amongst wheat varieties. WSVAS can quantify plant stress caused by salt stress and provides a powerful tool for salt-tolerant wheat breeding.

## 1 Introduction

Seeds are the beginning of the growth of each plant and the quality of crop seeds directly affects the yield of agricultural production. Wheat is one of the world’s major food crops and its physiological research has been a hot topic in agricultural research worldwide (Gao and Ayele, 2014; Das et al., 2017). In recent years, soil salinity caused by the extensive use of chemical fertilisers has become one of the main abiotic factors affecting the yield and quality degradation of wheat (Kanzari et al., 2012; Ranjbar and Jalali, 2016). Salt stress can cause excessive salt accumulation in plants, leading to ion toxicity, oxidative damage to the membrane system, osmoregulation imbalance and even plant death, thus seriously hindering the formation of wheat yield and quality (Zhou et al., 2021). Therefore, proper selection of salt-tolerant wheat varieties is crucial to meet the demand for wheat products globally.

Traditional seed germination tests usually employ methods to destroy seed samples and rely on manual measurements, which often limit the efficiency, scale and accuracy of the tests (Jahnke et al., 2016; RR Mir et al., 2015). Image processing techniques were first applied to seed vigour detection. Dell’Aquila et al. (2000) used image analysis to describe the water uptake characteristics of white kale seeds during germination and showed a linear relationship between seed area, perimeter and water content. Hoffmaster et al. (2003) developed an automated soybean seed vigour evaluation system using computer image processing techniques to evaluate soybean seed vigour indicators. Sako et al. (2001) successfully developed an automated seed viability imaging system (SVI) to accurately quantify seed viability through statistical data of morphological characteristics of lettuce seedling imaging. However, in the aforementioned studies, different light sources and acquisition environments greatly affect image quality, which makes simple image processing difficult (Anhua Ren et al., 2022; Xiuqing Fu et al., 2021).

In recent years, with the development of computer technology, the use of machine learning to detect seed vigour has become a hot topic in seed nondestructive testing (Zhe et al., 2020; M, Mladenov et al., 2019). Classical machine learning methods have various applications in seed vigour evaluation (Su et al, 2020; Bai et al, 2021). de Medeiros et al. (2020) combined image mapping and linear discriminant analysis models to predict seed germination. Awty-Carroll et al. (2018) used a KNN model to improve the scoring accuracy of mangosteen seeds. Lurstwut and Pornpanomchai (2017) developed a rice seed germination evaluation system (RSGES) based on an artificial neural network classifier to evaluate the germination status of Thai rice seeds. However, it proved that classical machine learning classifiers are based on manual feature extraction with slow training speed and low accuracy.

At present, convolutional neural networks (CNN) are increasingly and widely used for plant and animal image detection (Liu and Wang, 2020; Peng and Wang, 2022; Wang et al, 2022). The secondary detector represented by R-CNN for fast extraction and learning of image features was applied to the automated detection of batch images. Genze et al. (2020) used Faster R-CNN for seed germination detection and Yu et al. (2019) used mask-RCNN for fruit detection on picking robots. However, the disadvantage is that it is complex and requires high power consumption hardware devices. However, the first-level convolutional neural detectors represented by YOLO (you only look once) are faster in detection and have more significant lightweight features. The YOLO family of networks is constantly updated and improved in detection performance compared to other first-level object detectors such as SSD (Bochkovskiy et al., 2020).

This paper presents WSVAS, an accurate evaluation platform based on the CNN model YOLOv4 for automatic imaging of wheat seeds and rapid detection of germination. WSVAS integrates seed germination chamber hardware to control the seed culture experimental environment (e.g., temperature and light) and continuous acquisition of seed germination imaging, and a core algorithm based on the YOLOv4 CNN for detecting germinating wheat seeds and translating the image information into seed germination vigour under salt stress. With this platform, we have incubated and monitored four genotypes of seeds under three salt solutions, collected a series of real-time images of seed germination and analysed and quantified the germination profile plots of seeds under different salt stresses. WSVAS provides a powerful method to automatically evaluate plant salt tolerance by analysing the response of wheat seed germination to salt stress.

## 2 Materials and methods

### 2.1 Experiment design hardware equipment

**Figure 1:**
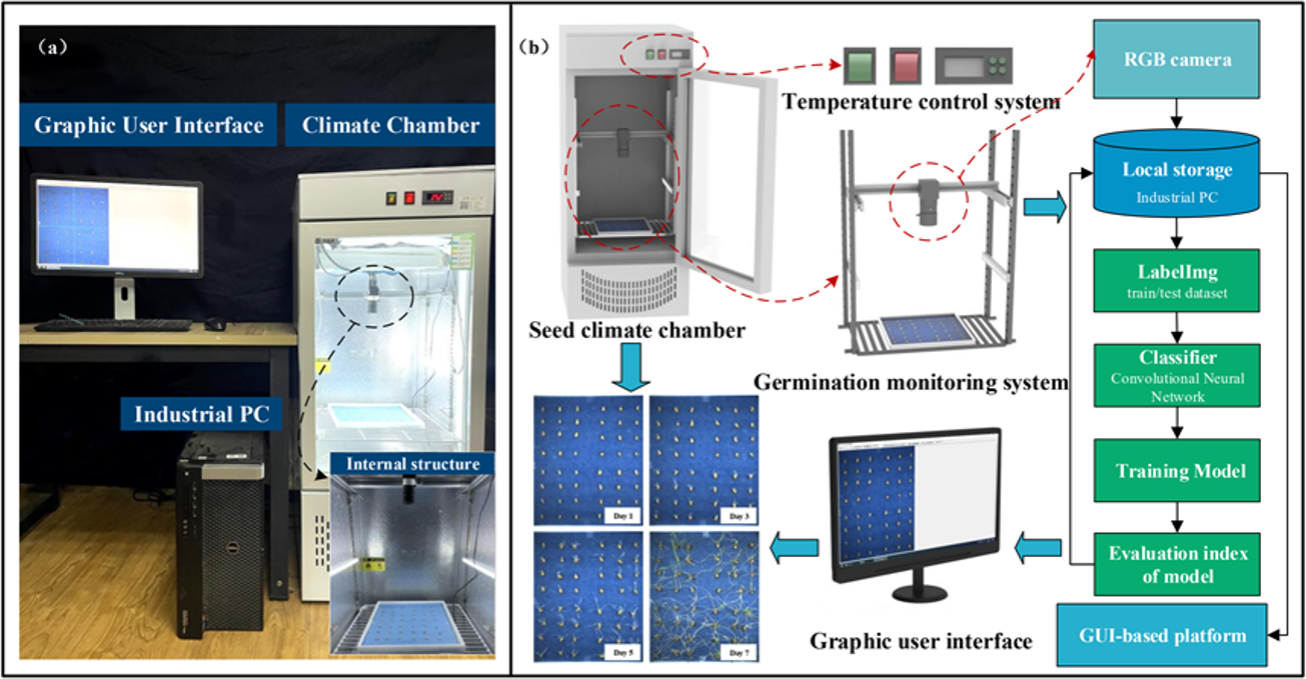
WSVAS hardware platform for real-time monitoring of seed germination.

WSVAS was designed for continuous monitoring of seed germination, as shown in **Figure 1**. The system consists of a seed chamber, an image-based real-time monitoring module and a control module. The seed chamber is used to contain and cultivate seeds in a controlled environment. Meanwhile, the seed germination chamber (ld - 100 l, Linde Intelligent Technologies Enterprise, Shandong, China), with a built-in PTC hot air circulation system and LED lamps, has an adjustable temperature range from 5°C to 50°C to germinate wide varieties of seeds. The WSVAS hardware platform is shown in **Figure 1A**: A complete set of hardware equipment, including seed germination climate chamber, image acquisition device - a miniature gantry structure with RBG camera on top for real-time monitoring of seed germination process. (b) Seed germination image acquisition process. The wheat seed germination test is conducted in a climate chamber. The temperature and humidity for seed germination and cultivation are set accordingly. The RGB camera is mounted on top of the germination box. Data is transmitted *via* the GigE connection. Seed germination images are displayed on the user interface and saved in local storage. It can be used for training neural network models in an industrial computer database. Finally, a series of single-group wheat seed germination test maps are created.

The image-processing-based monitoring module has an industrial camera (MV-HS510GC, Wei, Shanxi; M1224-MPW2, computer, Tokyo, Japan) with an image resolution of 2448 × 2048 pixels. **Figure 1B** shows the camera being mounted in a 40-cm-high micro-frame above the seed tray to capture seed images from a low-altitude view. The camera was configured to take images every 30 minutes to capture the entire process of seed germination. A series of images were transmitted to an edge computer (Microsoft Corporation; Redmond, WA, USA) *via* the camera’s gigabit network (GigE). The edge computer can immediately process the images according to the processing pipeline and visualise the results to the user for real-time monitoring.

### 2.2 Data acquisition and pre-processing

#### 2.2.1 Data collection scheme

**Figure 2:**
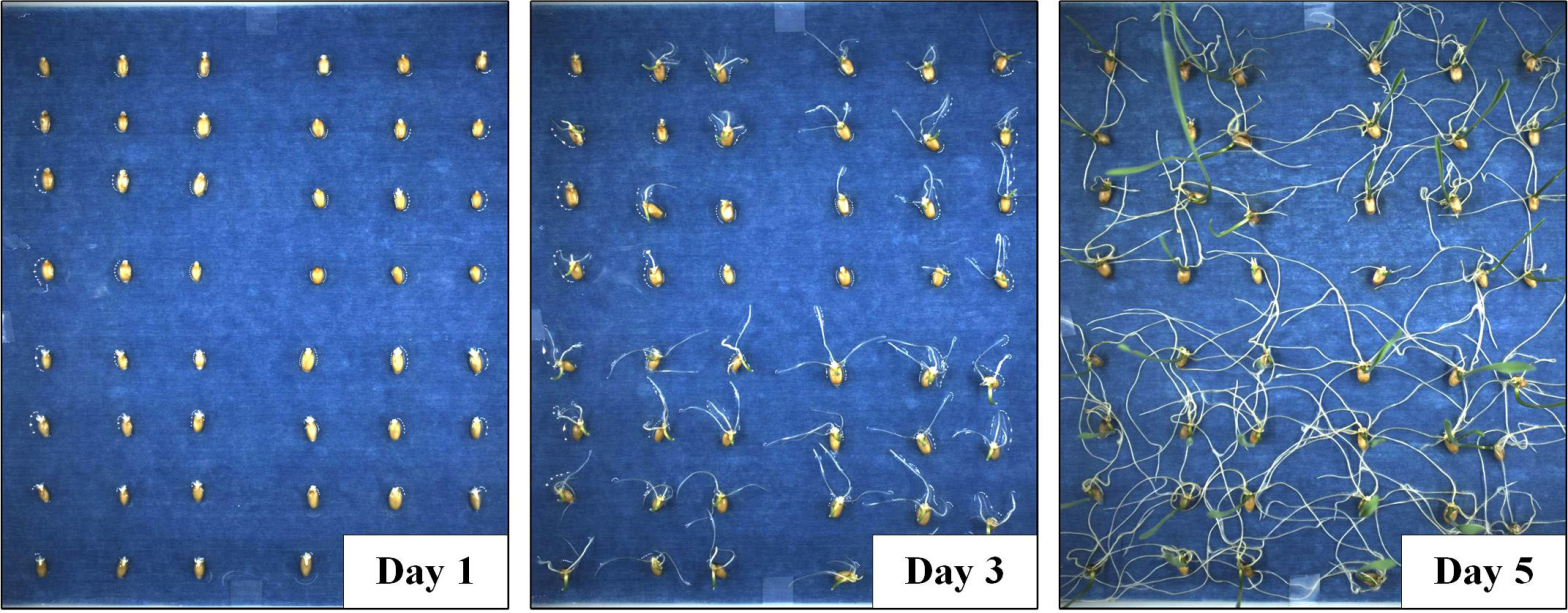
Continuous collection of growth images of wheat seeds during germination.

A total of 400 wheat seeds of uniform fullness and size were carefully selected, sterilised them with 2.5% sodium hypochlorite for 10 min, rinse them five times using distilled water and air-dry them in the shade afterwards. Then, the treated wheat seeds were soaked in distilled water for 12 h, the seeds were taken out to dry and placed on a blue filter paper waiting to be germinated. The bottom plate was a plastic tray with the size of 25 cm × 25 cm × 2 cm (acid and alkali resistant polyphenylene material), a total of 100 seeds were randomly placed on each tray and the same experiment was repeated four times. The seed germination Petri dishes were placed in a crop seed germination monitoring incubator with consistent environmental settings (24 h of light and 25°C room temperature). An RGB camera was set up on the developed user interface to take and collect pictures of all wheat seeds during germination every 30 minutes. Each experiment was conducted for 5 days and there were 48 images collected each day. A total of 960 images were collected during the entire experiment. All the captured images were saved in.jpg format on a local disk and the image resolutions were all 2448 × 2048 pixels, as shown in **Figure 2**. Meanwhile, Setting up salt stress experiments, four wheat varieties with different salt tolerance were selected as test materials, namely Huai Mai 33, Rotation 987, Zhen Mai 9 and Yang Mai 22. In addition, three sets of repeated tests were set to prevent test errors. Before the experiment, the wheat was firstly treated and then an equal number of seeds were subsequently placed in the given culture panels for different varieties. (Place 48 seeds in the given culture panel, including 4 wheat varieties, 12 seeds for each variety). There were three treatments applied in each culture panel under the same environmental settings: control (CK), 100 mmol/L salt stress (S1) and 150 mmol/L salt stress (S2).

#### 2.2.2 Data pre-processing

The data set used to train the seed germination detection model consisted of processed images and manual labels for each seed.

Data screening: Amongst the 960 images collected, there were images with severe root overlap of wheat seeds at the late germination stage, thereby affecting data labelling. After data cleaning, a total of 880 images were finally obtained for labelling.

Data augmentation: In deep learning training, data augmentation is used to expand the sample size to improve the robustness and generalisation ability of the model. The methods of data enhancement commonly used in target detection tasks are image scaling, flipping, cropping, colour transformation, adding noise, dithering and so on. Seed placement method and several seeds on model recognition accuracy, image cropping, random flip, and brightness transformation were used to perform offline data enhancement on 880 images to avoid the influence of brightness, as shown in **Figure S1**. There were a total of 1760 images after the enhancement.

**Figure 3:**
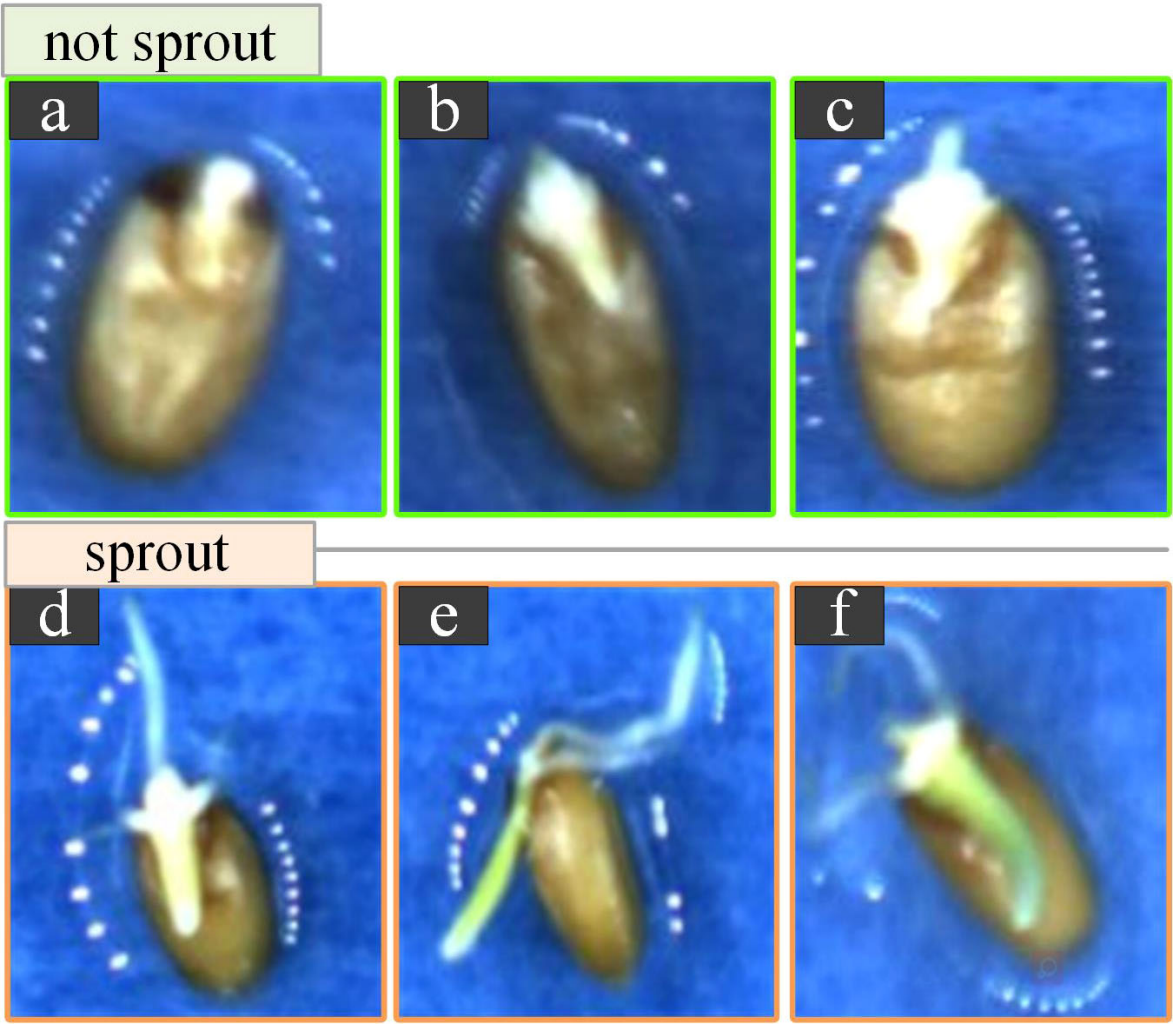
Schematic diagrams of germinated and ungerminated wheat seeds. **(A-C)**. Examples of ungerminated seeds. **(A)** Seeds are dewy with no germ radicle. **(B)**. There is a germ without a radicle. **(C)**. There is a germ radicle, however, it does not reach half of its length. **(D-F)**. Examples of germinated seeds. **(D)** Seeds have a radicle and the radicle has three distinct roots and it reaches half of its length. **(E)**. Seeds are lying on their sides. **(F)**. The radicle has taken on a green colour and is growing upward growth trend.

Data annotation: Manual annotation was performed using the open-source tool LabelImg. The labels were set to two types: “sprouting” and “not sprouting”. We considered wheat seeds germinating when the roots were visible and reached half the length of the seed itself, as shown in **Figure 3**. After tagging the images, a file with the file name ‘.xml’ was generated accordingly, which recorded the location of the tag box and the target category.

Data set partitioning: The data sets were randomly partitioned based on the ratio of 8:2, in which 80% of the training set was used for model training (a total of 1408 sheets) and 20% of the test set was used for model performance evaluation (a total of 352 sheets). During the training process, 10% of the validation set was divided into the training set for cross-validation to improve the model generalisation ability and prevent the occurrence of overfitting, which was used for the correction in model training (a total of 141 sheets). **Table 1** shows the information of the data set.

**Table 1:** Wheat seed data set information.

	Number of pictures	Category		Proportion
		Sprout	Unsprout	
Training set	1267	43763	73081	1:1.67
Validation set	141	4241	8853	1:2.09
Test set	352	12116	20162	1:1.66
Total	1760	60120	102096	1:1.70

### 2.3 CNN training

#### 2.3.1 Target detection model networks

**Figure 4:**
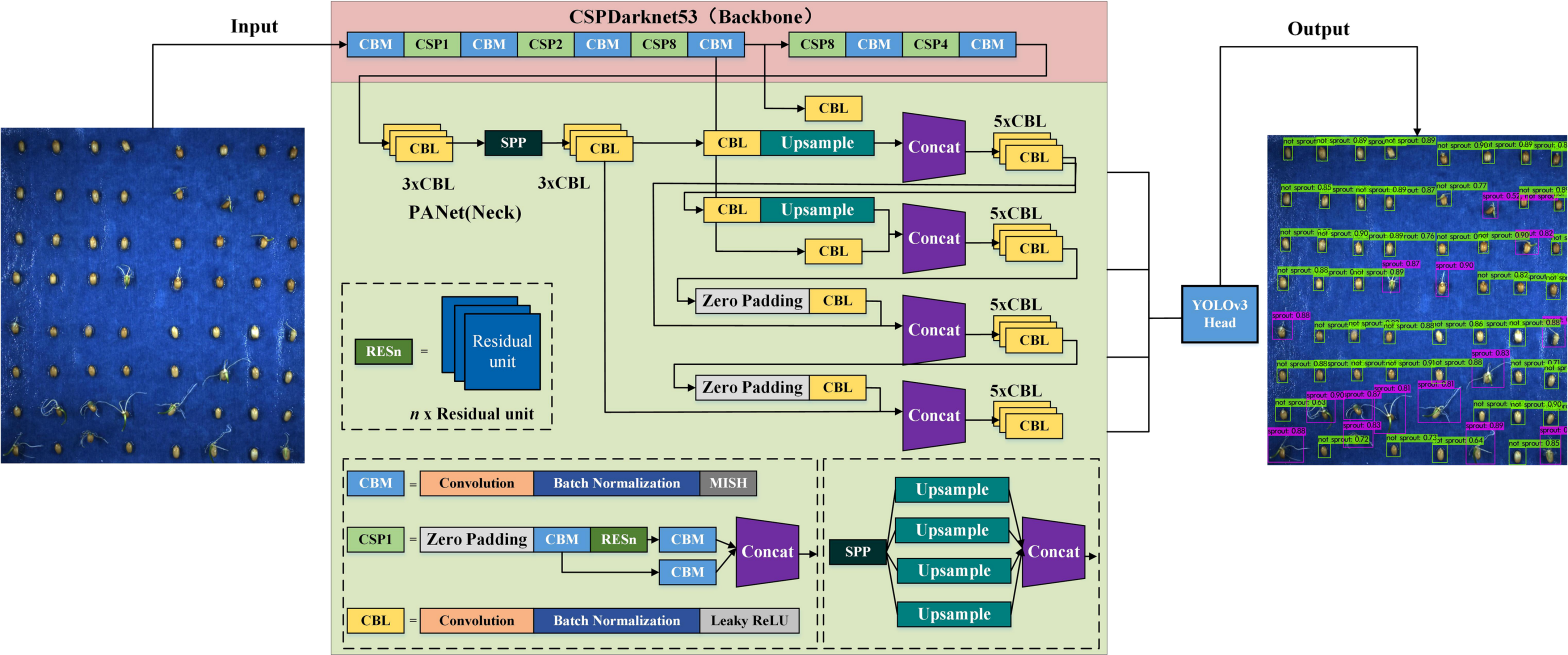
YOLOv4 network structure.

With the development of computer image technology, several neural networks for target detection and classification, such as YOLO, SSD, RetinaNet, Fast R-CNN and Faster R-CNN have been proposed. YOLO series is a classic representative of one stage detection algorithm, whose main idea is to use a single CNN to process images. The main idea is to use a single CNN to process the image and directly output the regression border coordinates and prediction categories thereby reducing the number of operations and increasing the detection speed. Therefore, YOLOv4, which has superior performance in small target detection and faster detection speed, is chosen in this study to meet the accuracy while satisfying real-time monitoring, and the model structure is shown in **Figure 4**.

We used CSPDarknet-53 as the backbone network, SPP as an additional module, PAN path aggregation network as the neck and YOLOv3 as the head to form the overall structure of YOLOv4 (Bochkovskiy et al, 2020; Liu et al, 2021). The model uses DarkNet-53 constructed by a residual network as the base network to deepen the network layers and reduce the problem of gradient loss or training degradation, thus enhancing the learning of image features and improving the recognition of germinated and ungerminated seeds (Suo et al, 2021).

#### 2.3.2 Model training and parameter design

Deep learning network model training usually requires a high configuration of the training platform, which can be trained on a CPU or GPU. Given that the computational power of GPU is higher and its training cost is lower than that of CPU, thus training on GPU is chosen. This study uses Windows 10 operating system, processor AMD Ryzen 9 5950X at 4.9GHz, graphics card GeForce RTX 3070, memory 64G, CUDA 10.1, cuDNN 7.6.5 and CMake 3.16 configured in Pycharm2019 development software. Using Python 3.8 freeze training can speed up the training efficiency and prevent the overfitting problem, we divide the whole training process into two phases, including the freeze training phase and unfreeze training phase. The hyperparameters include iteration number epoch, batch_size, learning rate, momentum, weight_ decay and so on. This model training mainly discusses the learning rate, batch_size and freezing epoch, the detailed discussion results are shown in schedule S1. Finally, different training parameters are adjusted to obtain the loss function change curve of the model. The untrained test set data are used to evaluate the performance of different models and compare the best detection model. The specific parameters are shown in **Table 2**.

**Table 2:** Training parameters of YOLO v4 wheat seed detection model.

Training stage	Parameter	Value
Freeze training stage	Input size	416x416
	Batch size	8
	Epochs	20
	Classes	2
	Optimiser	Adm
	Initial learning rate *l*_0_	1 × 10^-3^
	Minimum learning rate *l_min_*	1 × 10^-5^
	Decay	5 × 10^-4^
Post-thawing training stage	Input size	416 x 416
	Batch size	4
	Epochs	100
	Classes	2
	Optimiser	SGD
	Initial learning rate *l*_0_	5 × 10^-4^
	Minimum learning rate *l_min_*	5 × 10^-5^
	Decay	5 × 10^-4^

#### 2.3.3 Model evaluation metrics

**Figure 5:**
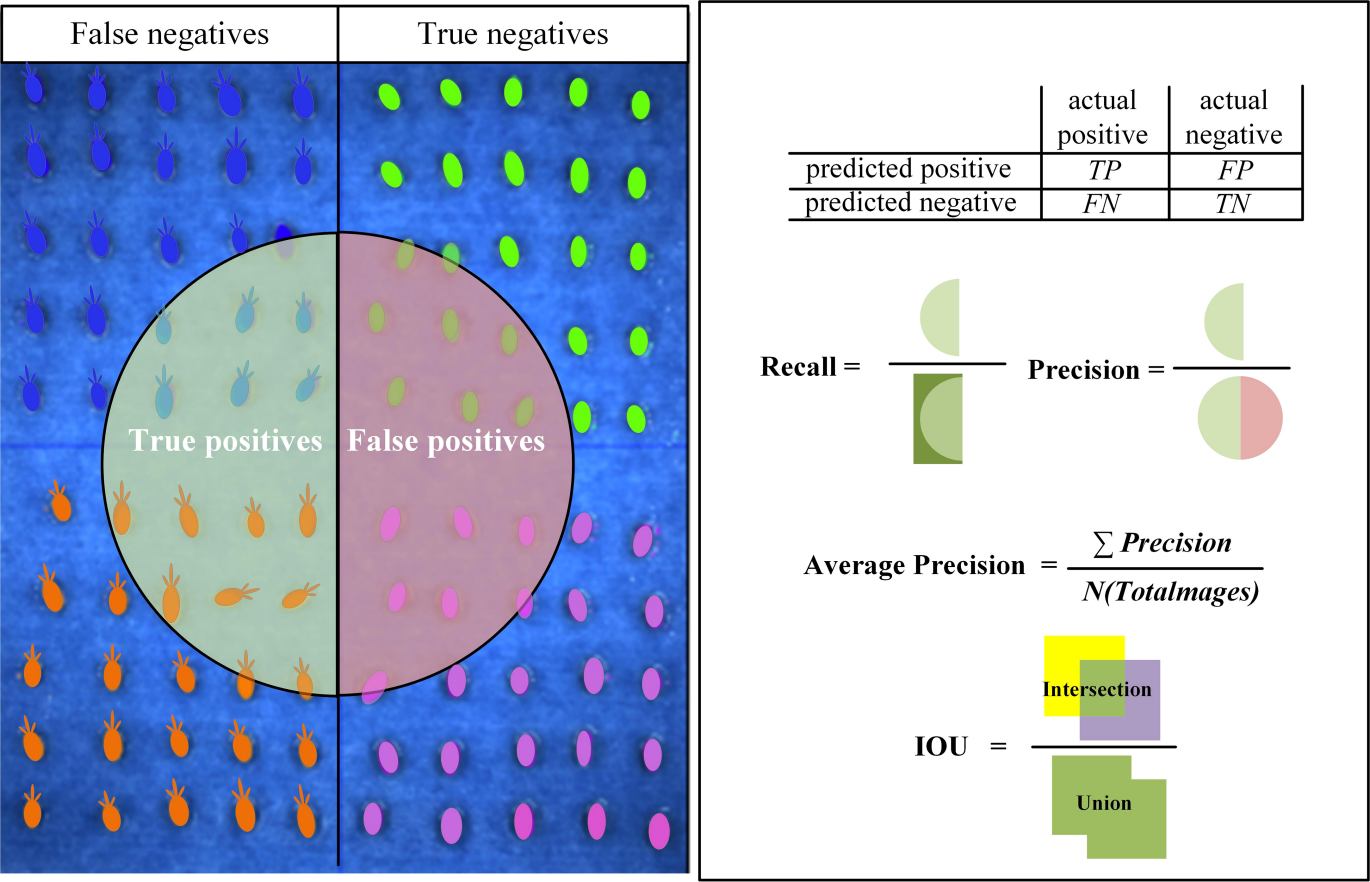
Evaluation indices of the model.

Accuracy (Precision), Recall (Recall), Average Precision (AP), mAP (mean Average Precision) and Intersection Over Union (IOU) are metrics widely used in the field of CNN to evaluate the quality of model training results. In this paper, we set “sprout” as positive and “not sprout” as negative in the target classification, and the four cases are shown in **Figure 5**.

The index evaluation of seed germination target identification results was calculated based on Equations (1–4).


(1) $$Precision=\frac{TP}{TP+FP}$$

(2) $$Recall=\frac{TP}{TP+FN}$$

(3) $$AP=\int_{0}^{1}p(r)dr$$

(4) $$IOU=\frac{p_{s}\cap^{}g_{s}}{p_{s}\cup^{}g_{s}}$$

where “p”, “r”, “p(r)”, “p_s_” and “g_s_” represent Precision, Recall, a parameter with a function of r, feature part of the seeds identified by the network model and features of the seeds tagged in the label, respectively.

#### 2.3.4 Indicators of germination vigour

Seed vigour is the sum of all characteristics that determine the activity and expression of the seed or seed germ during germination in various environments (Filho, 2015). Germination rate and germination index are commonly used in agriculture to detect seed vigour in crops. The calculation Equations are as follows (5):


(5) $$\text{Germination rate = }\frac{{\text{N}}_{\text{t}}}{\text{N}}\times 100\%$$

(6) $$\text{Germination index = }\frac{\sum {\text{G}}_{\text{t}}}{{\text{D}}_{\text{t}}}$$

where “Nt”, “N”, “Gt” and “Dt” denote the number of germinating seeds on the day “t”, the total number of seeds tested, the number of germinating seeds on the day “t” and seed growth period, respectively. Three biological replicates were measured for each treatment.

## 3 Results and discussion

### 3.1 Wheat germination identification and prediction

This study will compare the evaluation performance indices and detection effect plots under three models to select the best model for wheat seed germination target detection, VGG16 + Faster R-CNN, ResNet50 + Faster R-CNN and YOLO v4. **Table 3** shows that the YOLO v4-based wheat seed target detection model outperforms VGG16 + Faster R-CNN and ResNet50 + Faster R-CNN in terms of detection accuracy and speed, which coincides with the design requirements of high accuracy, high efficiency and low cost for wheat seed target detection system. (The performance parameters of the different detection models are shown in schedules S2 - S4)

**Table 3:** Comparison between detection accuracy and speed under three models.

Model	Input size	mAP	Recall	FPS
VGG16 + Faster R-CNN	600 × 600	90.38	86.33	2.41
ResNet50 + Faster R-CNN	600 × 600	93.52	90.17	1.61
YOLO v4	412 × 412	97.59	97.35	6.82

**Figure 6:**
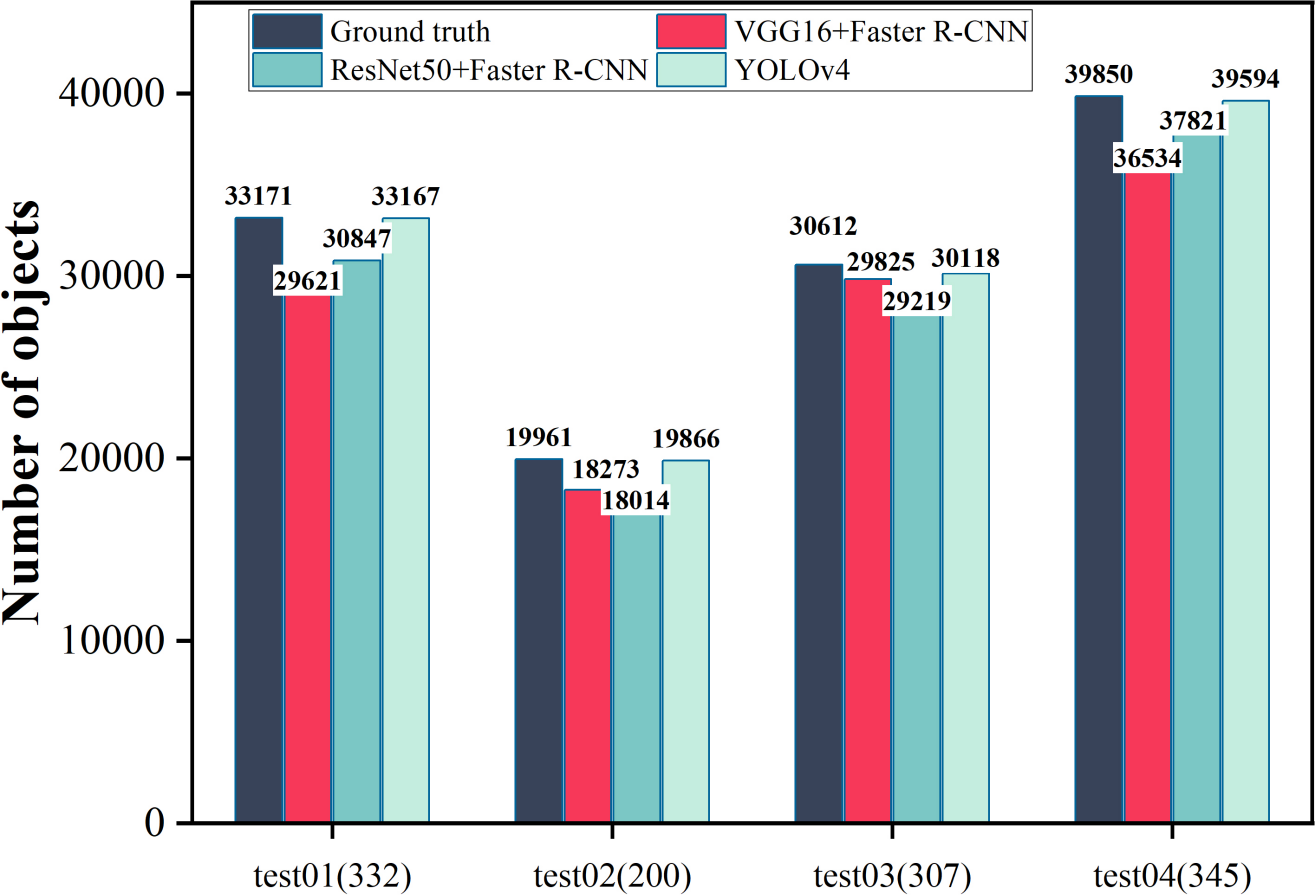
Comparison of the total number of detected targets for the three models on the four test sets. test01, test02, test03 and test04 represent the test set data of the early stage, middle stage, late stage and test set data of seed germination after data enhancement, respectively, to obtain the actual total number of germinating seed targets (GT, the Ground truth) and the total number of detected targets. The number of pictures of each test set is represented in parentheses.

In this study, the best iterative models under the training of three CNNs, VGG16 + Faster R-CNN, ResNet50 + Faster R-CNN and YOLOv4 were selected to test four different test sets, which represent the pre-, mid-, post-, and data-enhanced images of seed germination. **Figure 6** shows that in terms of the comparison between the total number of detected targets and the actual total number of targets, the YOLO v4-based seed germination target detection model is closer to the real situation than the other two models. Meanwhile, **Figure 6** also shows that in terms of test sets, the model is closer to the real value in terms of the number of targets detected in the middle and late stages of seed germination, and has higher accuracy in identifying germinating seeds and lower leakage rate and in terms of the data-enhanced data sets, the model showed a better detection performance. (A comparison of the total number of detected targets for each category with different model training parameters for YOLOv4 is shown in the attached **Figure S2**.)

**Figure 7:**
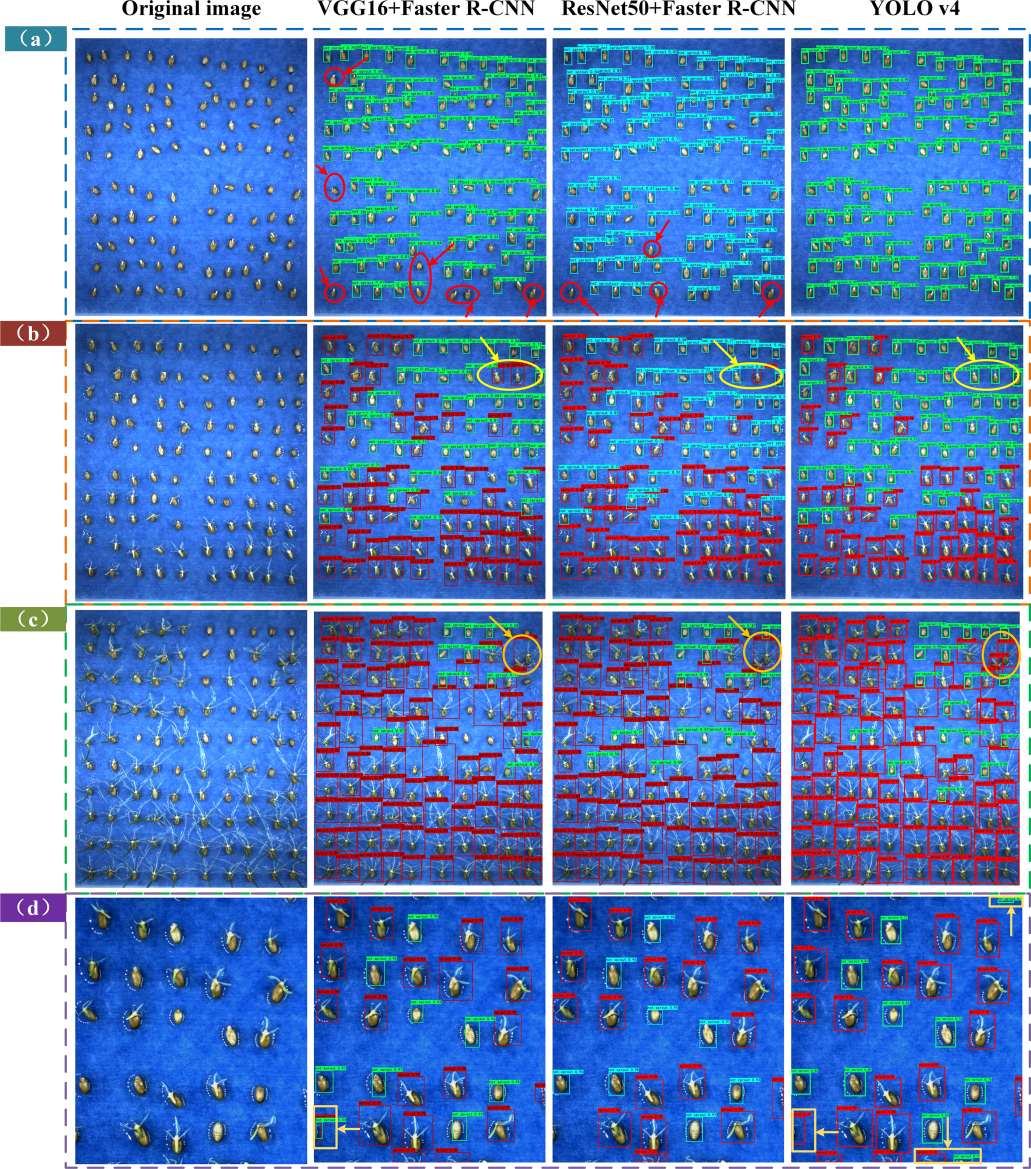
Comparison of the detection effect plots of the three models on the four test sets. **(A)** Test set test01 **(B)** Test set test02 **(C)** Test set test03 **(D)** Test set test04.

In **Figure 7A** in the test01 dataset, there were missed and under-detected cases in the VGG16 + Faster R-CNN and ResNet50 + Faster R-CNN based models, while in the YOLOv4 model, the ungerminated seeds were all detected. As shown in **Figure 7B** test02 dataset, the VGG16 + Faster R-CNN and ResNet50 + Faster R-CNN based models identified the unsprouted wheat seeds as sprouted and there was misclassification of the detection targets, while the YOLOv4 model exhibited correct classification. As shown in **Figure 7C**, the test03 dataset shows the different performance of the three models when the seed roots overlap in the late germination stage, the VGG16 + RPN and ResNet50 + Faster R-CNN models incorrectly classify the two seeds into one category, while the YOLOv4 model labels two different detection boxes for the two seeds and detects the number of target seeds closer to the actual labelled boxes.

As shown in **Figure 7D**, in the data-enhanced test04 dataset, the YOLOv4 model performs slightly worse than the VGG16 + Faster R-CNN and ResNet50 + Faster R-CNN models, which is reflected for targets with obscure seed features outside the images, the VGG16 + Faster R-CNN and ResNet50 + Faster R-CNN models are biased towards the detection of the seeds. Faster R-CNN models are biased towards no labelling, and the YOLOv4 model exhibits over-checking.

The correct detection rate, missed detection rate and over detection rate of the three models can be derived based on the above discussion, as shown in **Table 3**. As can be seen from the table, the YOLOv4-based wheat seed target detection model performs the best on the total test set, with a much higher correct rate than VGG16 + Faster R-CNN and ResNet50 + Faster R-CNN, and a lower miss detection rate than the latter two. However, the YOLOv4 model is too sensitive to seed features, and it recognises more seeds than the actual number of seeds in cropped wheat images and has a higher overdetection rate, which is almost 2–3 times higher than the VGG16 + Faster R-CNN and ResNet50 + Faster R-CNN models.

### 3.2 Wheat salt tolerance sprouting vigour detection

Seed germination is the beginning stage of plant growth and the most sensitive and vulnerable stage to salt stress (Fercha et al., 2014). Therefore, this experiment was conducted at the seed germination stage for salt stress to compare the salt tolerance of plants. Germination rate and germination index, as attributes of germination rate and more sensitive indicators of seed performance than cumulative germination percentage, can reflect the effect of salt stress on the germination rate of four wheat seeds with different salt tolerance genotypes. This reflects the differences in the performance of different genotypes of wheat seeds under salt stress and provides a basis for qualitative analysis of seed vigour.

**Figure 8:**
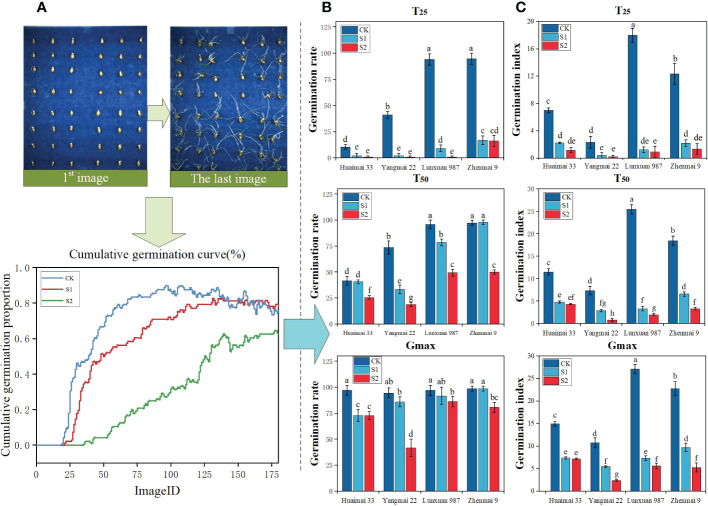
Results of germination vigour analysis of wheat seeds under different salt stresses. **(A)** A series of images and cumulative germination curves of the germination test process of wheat seeds under three groups of salt stress treatments **(B)** Quantitative germination rates, including the germination rates of wheat seeds of four genotypes at T25, T50 and Gmax stages under three groups of salt stress **(C)** Quantitative germination indices, including the germination indices of wheat seeds of four genotypes at T25, T50 and Gmax stages under three groups of salt stress. According to ANOVA and LSD, different letters indicate significant differences in specific sequences, *P*< 0.05.

Seeds under salt stress often show reduced germination and vigour and even death in severe cases, and such phenomena are mainly caused by water stress and ion imbalance (Çatav et al., 2021). **Figure 8A** reflects that the germination curve of wheat seeds showed a decreasing trend with increasing salt concentration, and the germination rate was CK > S1 > S2 in descending order, which is consistent with the results of previous studies (Zhu et al., 2021). **Figure 8B** reflects the decreasing trend of wheat seed germination with increasing salt concentration compared to the control. **Figure 8C** reflects the overall significant decrease of wheat seed germination index with increasing salinity in the salinity range from 0 to 150 mmol/L. There was a significant effect at the T25 stage of salt stress on the inhibition of germination vigour of wheat seeds of the four genotypes, where salt concentration in the T50 stage, the germination index of wheat under salt stress decreased significantly with the increase of salt concentration; in the Gmax stage, the germination indices of Huai Mai 33, Verticillium 987 and Zhen Mai 9 under salt stress all reached more than 5%, while that of Yang Mai 22 was only about 2.5%. The above results show that the salt stress significantly inhibited rosette 987 and town wheat 9 in the early germination stage, and all four genotypes of wheat were affected by salt stress in the middle germination stage. In addition, the germination rate of Yang wheat 22 was the lowest under salt stress in the late germination stage, followed by Huai wheat 33. (Sprouting vigour of different wheat varieties under salt stress is shown in schedule S5)

**Figure 9:**
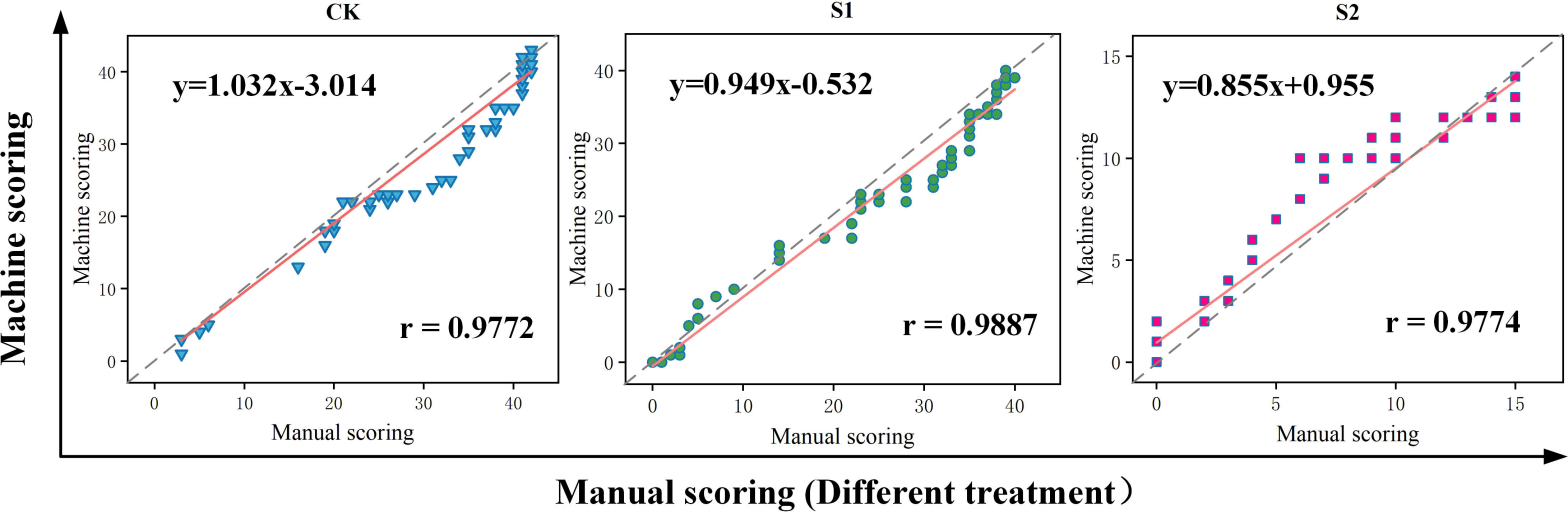
Linear correlation between manual scoring and machine scoring.

The variability of manual and platform counts was compared comprehensively to verify the accuracy of this seed germination platform. Three sets of trials were used to conduct manual counts according to image sequences (**Figure 9**). The cumulative number of germinations in each image and the image sequence during germination were recorded to compare the linear relationship between machine germination counts and manual counts as shown in **Figure 9**. **Figure 9** also includes the results of manual and machine scoring of seed germination numbers in three sets of trials, where the red line shows the deviation between manual and machine counts. The Pearson correlation index r for both manual and machine scoring in this trial reached a high level, thus indicating a good fit and a strong linear correlation. The highest degree of fit between manual and machine counts was achieved in the salt concentration of 100 mmol/L (treatment S1), with Pearson correlation index r reaching 0.9887, and the platform performed poorly in the salt concentration of 0 mmol/L (treatment CK) and salt concentration of 150 mmol/L (treatment S2), with Pearson correlation index r reaching only 0.9772 and 0.9774.

A large number of machine learning evaluation model metrics were used including mean square error (MSE), root mean square error (RMSE), mean absolute error (MAE), absolute coefficient (R2) and Pearson correlation metric (r). The results were generated as shown in **Table 4**. **Table 4** shows that the deviation between manual and machine counts in salt concentration of 100 mmol/L (treatment S1) was the smallest and the error was within a reasonable range, and the MAE and RMSE were 1.0066 and 1.3471, respectively, which were more accurate for detection. Then, was followed by a salt concentration of 0 mmol/L (treatment CK), and the worst detection accuracy of the platform under salt concentration of 150 mmol/L (treatment S2).

**Table 4:** Validation metrics used to compare manual counting and machine scoring.

Treatment	Pearson’s correlation r	R-Square	MAE	MSE	RMSE
CK	0.9772	0.9550	2.1287	8.3069	2.8822
S1	0.9887	0.9775	1.0066	1.8146	1.3471
S2	0.9774	0.9554	2.4040	10.5629	3.2501

## 4 Summary and prospect

In this study, a YOLOv4 CNN-based wheat seed salt tolerance germination vigour detection method was proposed to test the germination vigour of four different wheat species under three salt stresses and analyse the differences in salt tolerance of different wheat varieties to achieve an accurate assessment of wheat seed germination vigour under salt stress environment and real-time detection.

(1) The method uses YOLOv4 CNN to achieve accurate and rapid detection of a wheat seed number. In terms of model detection accuracy, the mean value of accuracy mAP and recall of the wheat seed germination detection model were 97.59% and 97.35%, respectively; in terms of detection speed, the speed of seed germination target detection model was designed based on YOLOv4 was as high as 6.82 frames per sec. Comparing the evaluation performance indices under the two models of YOLOv4 with VGG16 + Faster R-CNN and ResNet50 + Faster R-CNN, YOLOv4 outperforms the VGG16 + Faster R-CNN and ResNet50 + Faster R-CNN models in detection accuracy by 11.02% and 7.18%; in terms of detection speed, respectively. The seed germination target detection model designed based on YOLOv4 is the fastest, up to 6.82 frames/second. In addition, the experimental results show that different types of wheat seeds and salt stress environments do not greatly affect the quantitative detection results, indicating that this paper is feasible for accurate detection of wheat seeds by the lightweight CNN YOLOv4.

(2) The optimal detection model of wheat germinating seed target identification based on the YOLOv4 network was used to count the number of germinating seeds in the pictures. The germination curve of wheat seed germination number with time was plotted, and the germination index and germination rate of wheat under salt concentration were obtained and then the effect of salt stress on the germination vigour of different genotypes of wheat seeds was analysed. The differences between the number of germination and manual counts based on the crop seed germination monitoring system were also compared. A linear regression analysis was performed to provide technical support for the establishment of seed germination vigour ratings and wheat seed breeding for salt tolerance. In addition, this study can provide some technical references for studying the germination vigour of more seeds and establish a perfect seed vigour rating model.

WSVAS can meet more parameters of detection by further developing and optimising different users’ collection needs, which provides a powerful tool for studying diverse seed early growth performance and predicting future yield with high process value.

## Data availability statement

The original contributions presented in the study are included in the article/**Supplementary Material**. Further inquiries can be directed to the corresponding author.

## Author contributions

XF: Conceptualization, Data curation, Software. BH: Software, Validation, Writing-original draft. SL: Supervision, Investigation, Resources. JZ: Supervision, Methodology, Resources. HWZ: Supervision, Investigation, Resources. HW: Supervision, Investigation, Resources. HZ: Supervision, Investigation, Resources. ZO: Supervision, Investigation, Resources. All authors contributed to the article and approved the submitted version.
