# Supplementary material for: WSVAS: A YOLOv4 -based phenotyping platform for automatically detecting the salt tolerance of wheat based on seed germination vigour
Source: Front Plant Sci. 2022 Dec 20;13:1074360. doi: 10.3389/fpls.2022.1074360 (PMC9807913; doi:10.3389/fpls.2022.1074360)
Supplement: Supplementary file 1 [file DataSheet_1.docx]

Supplementary Material

# Supplementary Figures and Tables

## Supplementary Figures


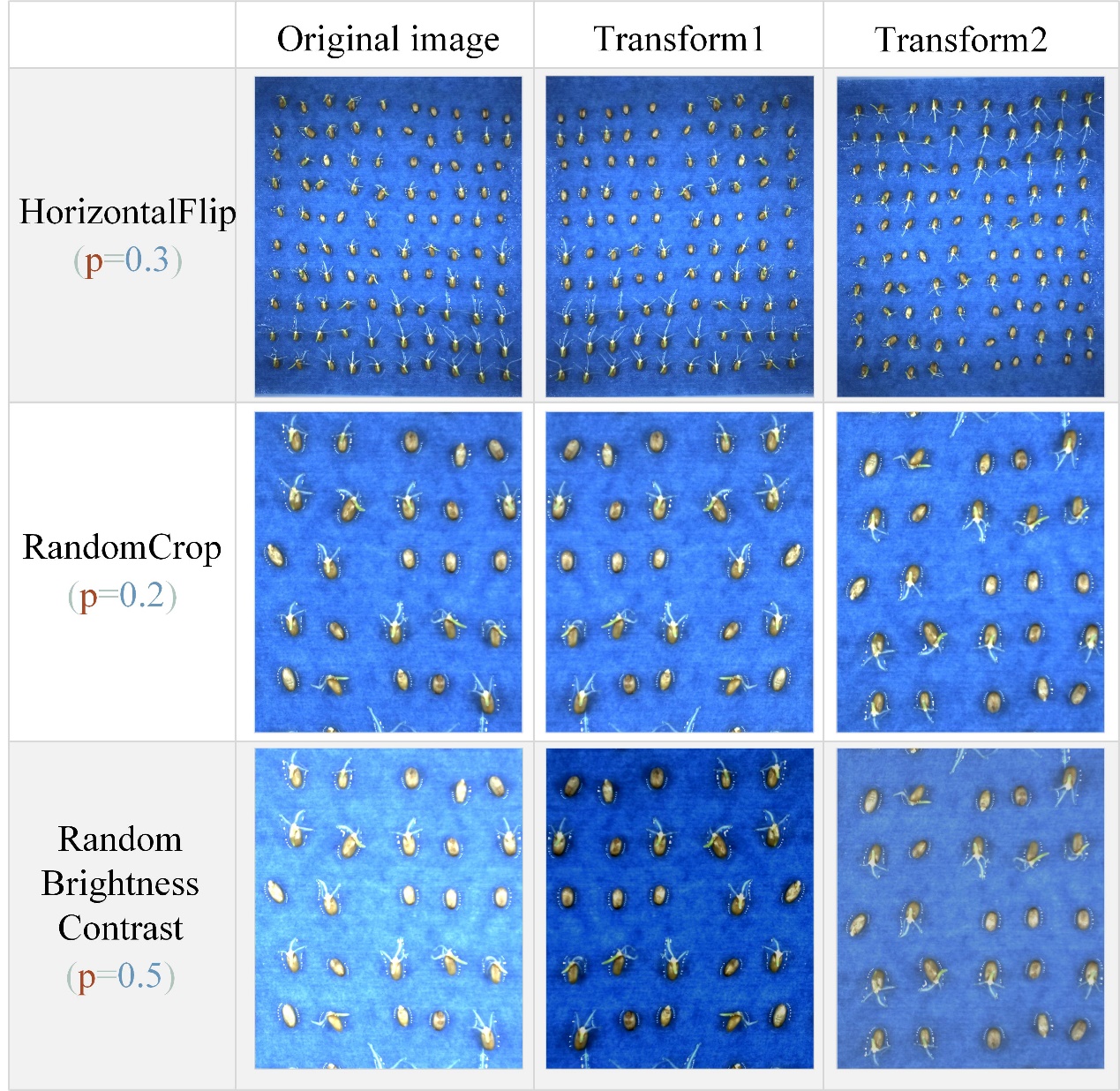


Supplementary Figure S1 Data enhancement process: To define the enhancement channels, first, create the Compose class, which receives a list containing three data enhancement functions: A.HorizontalFlip, A.RandomCrop and A.RandomBrighntessContrast, where A.HorizontalFlip is used to randomly flip the image horizontally, A.RandomCrop is used to randomly crop the image and A.RandomBrighntessContrast is used to randomly transform the brightness. The parameter p controls the probability of performing the enhancement, e.g. p=0.3 means there is a 30% probability of flipping the image horizontally and a 70% probability that the image will not change. The image is read from the local disk, the image is transformed, the enhanced image is displayed on the visualisation interface and finally saved to the storage directory.

Supplementary Figure. S2 Plot of the total number of detected targets versus the actual total number of targets for each category with different model training parameters for YOLOv4: not sprout-gt indicates the total number of category unsprouted targets, not sprout-fp indicates the number of detected target error positive classes, not sprout-tp indicates the number of detected target correct positive classes, sprout-gt indicates the total number of category sprouted targets, sprout-fp indicates the number of detected target error positive classes, and sprout-tp indicates the number of detected target correct positive classes.

## Supplementary Tables

Supplementary Table S1 Table of training parameters for the YOLOv4 model

| model | **AP（%）** | | mAP（%） | **Recall（%）** | | **F1（%）** |
| --- | --- | --- | --- | --- | --- | --- |
|  | sprout | not sprout |  | sprout | not sprout |  |
| 1 | 97.34 | 97.25 | 97.30 | 97.74 | 97.03 | 97 |
| 2 | 96.39 | 96.99 | 96.69 | 96.93 | 96.63 | 96 |
| 3 | 96.58 | 97.51 | 97.05 | 97.25 | 96.92 | 97 |
| 4 | 97.22 | 97.95 | 97.59 | 97.42 | 97.28 | 97 |
| 5 | 96.72 | 97.62 | 97.17 | 96.53 | 97.25 | 96 |
| 6 | 97.38 | 96.79 | 97.09 | 97.26 | 96.75 | 97 |
| 7 | 97.14 | 97.02 | 97.08 | 97.38 | 97.14 | 97 |
| 8 | 97.29 | 97.06 | 97.18 | 97.47 | 97.18 | 97 |
| 9 | 97.40 | 97.04 | 97.22 | 97.96 | 97.26 | 97 |

Supplementary Table S2 Table of performance parameters of VGG16 detection model

| Model（VGG16） | Model_100 | | Model_120 | | Model_140 | |
| --- | --- | --- | --- | --- | --- | --- |
| p=precision, r=recall, c=confidence, u=nms_iou | sprout | not sprout | sprout | not sprout | sprout | not sprout |
| (p)mAP=0.50 \|c=0.5, u=0.3 | 95.31 | 85.71 | 95.35 | 85.40 | 95.31 | 85.62 |
| (p)mAP=0.50 \|c=0.5, u=0.5 | 94.91 | 81.00 | 94.87 | 81.03 | 94.86 | 81.23 |
| (p)mAP=0.65 \|c=0.5, u=0.3 | 75.63 | 66.12 | 75.16 | 66.28 | 75.24 | 66.12 |
| (r)mAP=0.50 \|c=0.5, u=0.3 | 95.17 | 76.79 | 95.38 | 77.28 | 95.33 | 77.18 |
| (r)mAP=0.50 \|c=0.5, u=0.5 | 95.34 | 78.11 | 95.53 | 78.72 | 95.46 | 78.56 |
| (r)mAP=0.65 \|c=0.5, u=0.3 | 76.01 | 56.52 | 75.69 | 57.12 | 75.71 | 56.76 |

Supplementary Table S3 Table of performance parameters of ResNet50 detection model

| Model（ResNet50） | Model_100 | | Model_120 | | Model_140 | | |
| --- | --- | --- | --- | --- | --- | --- | --- |
| p=precision, r=recall, c=confidence, u=nms_iou | sprout | not sprout | sprout | not sprout | sprout | not sprout | |
| (p)mAP=0.50 \|c=0.5, u=0.3 | 96.29 | 90.49 | 96.31 | 90.72 | 96.36 | 90.68 | |
| (p)mAP=0.50 \|c=0.5, u=0.5 | 96.08 | 86.21 | 96.19 | 86.30 | 96.31 | 86.27 | |
| (p)mAP=0.65 \|c=0.5, u=0.3 | 83.10 | 74.18 | 83.42 | 74.42 | 83.29 | 74.51 | |
| (r)mAP=0.50 \|c=0.5, u=0.3 | 96.14 | 84.02 | 96.05 | 84.28 | 96.06 | 84.29 | |
| (r)mAP=0.50 \|c=0.5, u=0.5 | 96.30 | 85.14 | 96.28 | 85.38 | 96.31 | 85.40 | |
| (r)mAP=0.65 \|c=0.5, u=0.3 | 83.30 | 66.19 | 83.50 | 66.54 | 83.36 | | 66.63 |

Supplementary Table S5 Germination vigor of different wheat varieties under salt stress

| Germination period | Varieties | Treatment | Germination rate | Germination index |
| --- | --- | --- | --- | --- |
| T_25_ | *Huaimai33* | CK | 10.40±2.10^d^ | 6.99±0.36^C^ |
|  |  | S1 | 2.08±2.09^e^ | 2.24±0.13^D^ |
|  |  | S2 | 0.69±1.20^e^ | 1.15±0.38^DE^ |
|  | *Yangmai22* | CK | 40.98±3.19^b^ | 2.33±0.87^D^ |
|  |  | S1 | 2.08±2.09^e^ | 0.39±0.42^E^ |
|  |  | S2 | 0.69±1.20^e^ | 0.22±0.19^E^ |
|  | *Lunxuan987* | CK | 93.75±5.21^a^ | 18.00±1.00^A^ |
|  |  | S1 | 9.02±3.19^d^ | 1.22±0.42^DE^ |
|  |  | S2 | 0.69±1.20^e^ | 0.89±0.84^E^ |
|  | *Zhenmai9* | CK | 94.44±5.24^a^ | 12.33±1.53^B^ |
|  |  | S1 | 16.68±4.17^c^ | 2.17±0.50^D^ |
|  |  | S2 | 16.00±5.21^cd^ | 1.34±0.76^DE^ |
| T_50_ | *Huaimai33* | CK | 41.63±4.15^d^ | 11.48±0.70^C^ |
|  |  | S1 | 40.61±1.89^d^ | 4.79±0.24^E^ |
|  |  | S2 | 25.61±1.91^f^ | 4.29±0.15^EF^ |
|  | *Yangmai22* | CK | 73.61±6.36^b^ | 7.31±0.92^D^ |
|  |  | S1 | 33.32±4.17^e^ | 2.86±0.26^FG^ |
|  |  | S2 | 18.76±2.06^g^ | 0.77±0.42^H^ |
|  | *Lunxuan987* | CK | 95.82±4.16^a^ | 25.43±1.05^A^ |
|  |  | S1 | 78.47±3.18^b^ | 3.29±0.51^F^ |
|  |  | S2 | 49.30±3.18^c^ | 1.94±0.23^G^ |
|  | *Zhenmai9* | CK | 97.20±2.42^a^ | 18.40±1.05^B^ |
|  |  | S1 | 97.90±2.10^a^ | 6.55±0.48^D^ |
|  |  | S2 | 50.00±2.08^c^ | 3.30±0.30^F^ |
| G_max_ | *Huaimai33* | CK | 97.23±4.79^a^ | 14.96±0.49^C^ |
|  |  | S1 | 72.75±5.85^c^ | 7.40±0.26^E^ |
|  |  | S2 | 72.76±3.87^c^ | 7.21±0.16^E^ |
|  | *Yangmai22* | CK | 94.47±4.79^ab^ | 10.75±1.07^A^ |
|  |  | S1 | 86.07±4.79^b^ | 5.44±0.22^F^ |
|  |  | S2 | 41.67±8.35^d^ | 2.35±0.22^G^ |
|  | *Lunxuan987* | CK | 97.22±4.81^a^ | 27.12±1.02^A^ |
|  |  | S1 | 91.66±8.35^ab^ | 7.31±0.51^E^ |
|  |  | S2 | 86.09±4.83^b^ | 5.61±0.59^F^ |
|  | *Zhenmai9* | CK | 98.60±2.42^a^ | 22.79±1.51^G^ |
|  |  | S1 | 98.60±2.42^a^ | 9.70±0.92^E^ |
|  |  | S2 | 80.53±4.79^bc^ | 5.27±1.03^F^ |
